# O.4.5-3 Trend in sedentary behaviour in the Netherlands

**DOI:** 10.1093/eurpub/ckad133.211

**Published:** 2023-09-11

**Authors:** Tessa Schurink-van 't Klooster, Sacha van Deemter, Marjolein Duijvestijn, Wanda Wendel-Vos

**Affiliations:** National Institute for Public Health and the environment (RIVM), The Netherlands; marjolein.duijvestijn@rivm.nl; National Institute for Public Health and the environment (RIVM), The Netherlands; marjolein.duijvestijn@rivm.nl; National Institute for Public Health and the environment (RIVM), The Netherlands; marjolein.duijvestijn@rivm.nl; National Institute for Public Health and the environment (RIVM), The Netherlands; marjolein.duijvestijn@rivm.nl

## Abstract

**Purpose:**

Prolonged sedentary behaviour has been associated with a higher risk of cardio-vascular diseases and premature death. The general recommendation is to interrupt long periods of sitting. The aim of this study was to investigate trends in sedentary behaviour among (subgroups of) the Dutch population.

**Methods:**

Data from the Additional module Physical activity and Accidents of the Dutch Lifestyle Monitor was used, which includes a representative sample of the Dutch population aged 4 years and older. Sedentary time was measured in the years 2015, 2017, 2019 and 2021, using an adjusted version of the Marshall questionnaire. Sitting domains were: 1) traveling, 2) at work, 3) at school or studying 4) watching television, 5) using a computer/smartphone at home, and 6) otherwise. Trends in sedentary time on an average day of the week, in total and per domain, were analysed for the total Dutch population and stratified by different background characteristics using univariate and multivariate linear regression analysis.

**Results:**

A positive (i.e. increasing) trend over time was seen in the number of hours spent sitting, i.e. from 8.7 hours in 2015 to 9.1 hours in 2021 (p = 0.003). In the following groups this trend was more increasing compared to other groups of the particular background characteristic: women, older age groups (65- to 79-year-olds and people over 80), lower educated people, people with an household income in the 2^nd^ quintile, partners in a couple with or without children living at home, paid workers working ≥32 hours per week, people in good health, overweight and obese persons, people who did not meet the Physical Activity Guidelines, and people who did not exercise weekly. These trends were mostly seen in the domains: at school or studying, watching television, and other leisure time activities.

**Conclusions:**

Our study showed an increasing trend between 2015 and 2021 in sedentary behaviour in several groups of the Dutch population of 4 years and older. These results provide possible starting points for future policy, but can also be used as a benchmark for other studies of sedentary behaviour among different target groups.

